# Comparative expression analysis of water buffalo (*Bubalus bubalis*) to identify genes associated with economically important traits

**DOI:** 10.3389/fvets.2023.1160486

**Published:** 2023-05-12

**Authors:** Dwijesh Chandra Mishra, Jyotika Bhati, Sunita Yadav, Himanshu Avashthi, Poonam Sikka, Andonissamy Jerome, Ashok Kumar Balhara, Inderjeet Singh, Anil Rai, Krishna Kumar Chaturvedi

**Affiliations:** ^1^ICAR-Indian Agricultural Statistics Research Institute, Indian Council of Agricultural Research (ICAR), PUSA, New Delhi, India; ^2^ICAR-Central Institute for Research on Buffaloes, Indian Council of Agricultural Research (ICAR), Hisar, India

**Keywords:** water buffalo, transcriptome, annotation, GO terms, SSRs

## Abstract

The milk, meat, skins, and draft power of domestic water buffalo (*Bubalus bubalis*) provide substantial contributions to the global agricultural economy. The world's water buffalo population is primarily found in Asia, and the buffalo supports more people per capita than any other livestock species. For evaluating the workflow, output rate, and completeness of transcriptome assemblies within and between reference-free (RF) *de novo* transcriptome and reference-based (RB) datasets, abundant bioinformatics studies have been carried out to date. However, comprehensive documentation of the degree of consistency and variability of the data produced by comparing gene expression levels using these two separate techniques is lacking. In the present study, we assessed the variations in the number of differentially expressed genes (DEGs) attained with RF and RB approaches. In light of this, we conducted a study to identify, annotate, and analyze the genes associated with four economically important traits of buffalo, *viz*., milk volume, age at first calving, post-partum cyclicity, and feed conversion efficiency. A total of 14,201 and 279 DEGs were identified in RF and RB assemblies. Gene ontology (GO) terms associated with the identified genes were allocated to traits under study. Identified genes improve the knowledge of the underlying mechanism of trait expression in water buffalo which may support improved breeding plans for higher productivity. The empirical findings of this study using RNA-seq data-based assembly may improve the understanding of genetic diversity in relation to buffalo productivity and provide important contributions to answer biological issues regarding the transcriptome of non-model organisms.

## Introduction

The domestic water buffalo (*Bubalus bubalis*) marks a key impact on the global agricultural economy through milk, meat, and draft power. The world's water buffalo population is largely found in Asia, and most people consider it the most promising livestock species for their livelihood (1, 2). Asia accounts for 97% of the total buffalo production with the largest population in India (>100 million) (2). More than half of the milk produced in India comes from buffaloes, which also produce milk with higher levels of fat, particularly saturated fatty acids, than cattle (2). Buffaloes are resilient to the harsher environment and resistant to several bovine tropical diseases (1), thus may have better feed convergence while surviving on poor-quality roughage than cattle. Recent studies cataloging differentially expressed genes (DEGs) and variants (3, 4) with respect to important performance traits in water buffalo corroborate with the functional genetic diversity in this species.

Milk volume, age at first calving, post-partum cyclicity, and feed conversion efficiency traits define the overall productivity of buffaloes. Buffalo milk has the significance of having higher concentrations of fat, lactose, protein, ash, calcium, and vitamins A and C while having lower concentrations of cholesterol and the blue-green pigment (biliverdin) (5). Additionally, buffalo milk has bioactive pentasaccharides and gangliosides, which are absent in cow milk (6). Therefore, the study of genes related to milk volume is very important. The age at first calving can be used to determine a buffalo's fertility and productivity. Buffalo productivity is affected by delayed puberty onset and inadequate consecutive estrus detection (7). Reproductive efficiency is the primary factor affecting the productivity of buffaloes, which comprise early age at first calving (AFC) and optimum service period between the calvings (post-partum cyclicity) throughout the reproductive span in life. Thus, the identification of genes and the variants associated with these traits may support selective breeding for genetic improvement. Improved immunity is equally significant to propagate uterine cleansing to facilitate an early resumption of ovarian cyclicity (8, 9). Feed conversion efficiency (FCE) is defined as a dry matter intake (DMI) per unit body weight (g/day) gain determined as residual feed intake (RFI). It represents the difference in actual and predicted DMI of each individual heifer (10). Feed conversion efficiency is a heritable trait governed by common biomolecules as growth hormones are associated with milk volume, age at first calving, and post-partum cyclicity (11, 12).

Several studies have confirmed the discovery of differentially expressed as well as novel genes in mammals such as humans, buffaloes, sheep, goats, and pigs (3, 13–17). The genetic link and diversity among various buffalo breeds have primarily been studied using restriction fragment length polymorphism (RFLP) (18), random amplified polymorphic DNA (RAPD) (19), single nucleotide polymorphism (SNP) (4, 20), and simple sequence repeat (SSR) (21) markers. SSR markers have proven to be an incredibly powerful tool for researching genetic divergence and/or genetic resource conservation (22). Identification and characterization of genome-wide DEGs related to reproduction and production traits can be widely used for selective breeding, which may enhance productivity in buffaloes (23, 24).

Considering this, an attempt was made to identify the variants related to important traits, i.e., milk volume, age at first calving, post-partum cyclicity, and feed conversion efficiency, using transcriptomic data to improve breeding plans in water buffaloes. DEGs were identified, characterized, and annotated, in order to accelerate performance in buffaloes through molecular breeding. This is a unique study identifying the functional classifications of genes, variants, and SSRs related to desired traits in *B. bubalis*.

## Materials and methods

**Figure 1:**
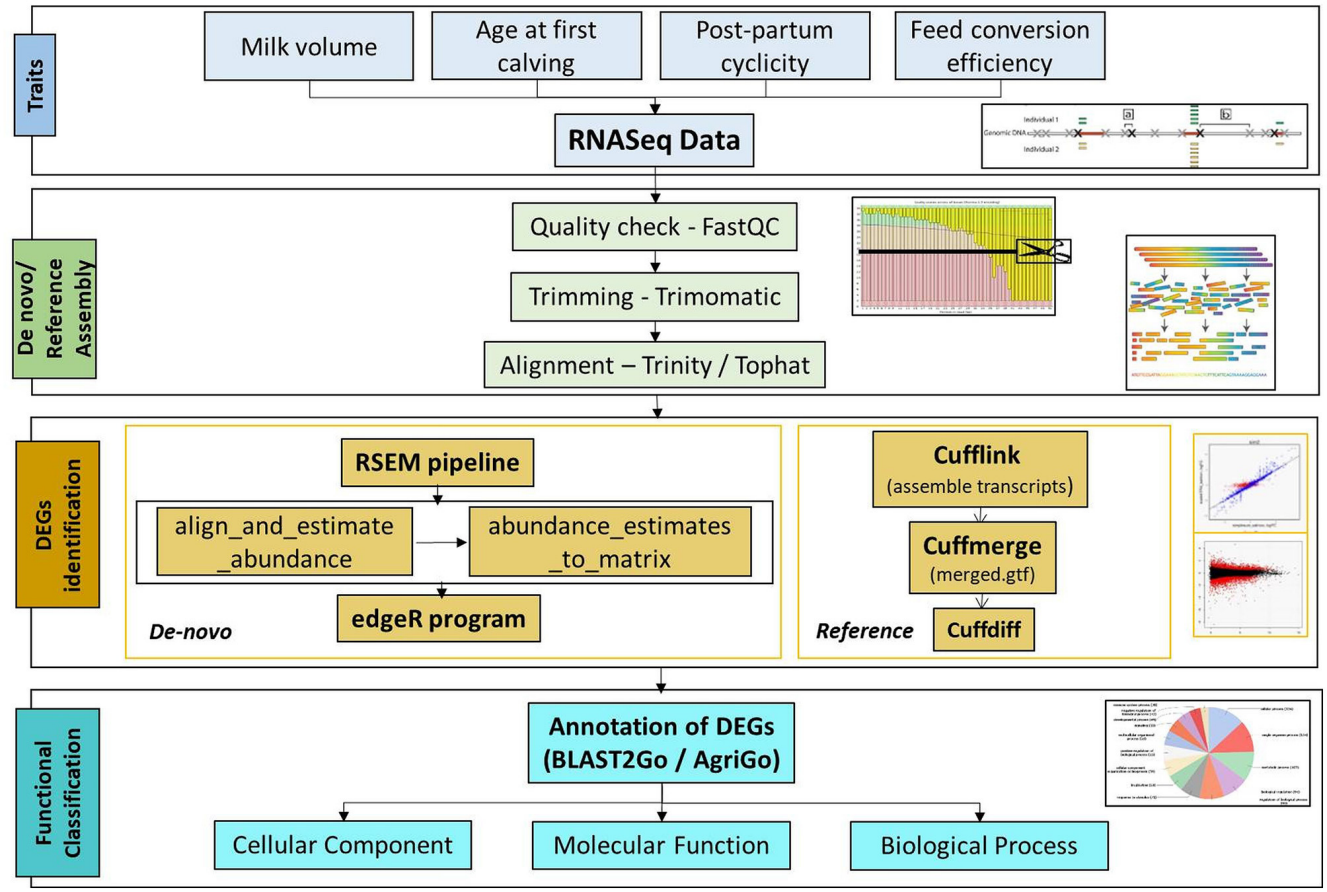
Workflow of the RNAseq data analysis.

Milk volume, age at first calving, post-partum cyclicity, and feed conversion efficiency were the four different traits for which datasets were collected. Four samples were selected for each trait (two each of low and high expression). The complete workflow of the study is presented in Figure 1.

### Ethics statement

Animals (*n* = 16) were selected as per the referred design for selective genotyping of buffaloes, based on performance phenotype recorded at ICAR-Central Institute for Research on Buffaloes (ICAR-CIRB), Hisar, Haryana, India. Genotype data were generated for selected genotypes through outsourced services hired by the institute. Animals were maintained under farm management at the institute, and the experiment design was approved by the Institute Animal Ethics Committee (IAEC) with ethics approval number−406/GO/RBI/L/01/CPCSEA.

### Animals and tissue collection

Whole blood tissues of individual animals were selected from unrelated pedigrees having extreme performance levels for complex traits as follows: milk volume, age at first calving, post-partum cyclicity, and feed conversion efficiency. Each trait had four samples comprising two each of low and high expressions.

### RNA extraction, library preparation, and sequencing

For the transcriptome analyses of expression patterns in low- and high-performing Murrah buffaloes, cDNA was generated using a routine RNA library preparation HiSeq protocol developed by Illumina Technologies (San Diego, CA), using 1 μg of total RNA as input. Using the High-Capacity cDNA Reverse Transcription kit (Life Technologies, Frederick, Maryland, USA), mRNA was first isolated from total RNA by performing a polyA selection step, followed by the construction of paired-end sequencing libraries with an insert size of ~300 bp. In brief, polyA selected RNA was cleaved as per Illumina protocol, and the cleaved fragments were used to generate first-strand cDNA using Super Script II reverse transcriptase and random hexamers. Subsequently, the second strand cDNA was synthesized with RNaseH and DNA polymerase enzyme, followed by adapter ligation and end-repair steps. The resulting products were amplified via PCR, and cDNA libraries were then purified and validated using the Agilent 2200 Tape Station system (Agilent Technologies Brasil Ltda, São Paulo, SP, Brazil). Paired-end sequencing was performed using the Illumina HiScanSQ platform. Samples were multiplexed with unique hexamer barcodes and run on multiple lanes to obtain 2 × 100 bp reads. Paired-end FASTQ files were subjected to standard quality control based on Phred scores of >20, using the NGSQC Tool kit v2.2 (25) to obtain high-quality (HQ) filtered reads.

### Transcriptome assembly and optimization

Raw reads from the four sets generated from the animal samples using Illumina HiSeq were filtered to generate clean data to remove adaptor sequences, reads with ambiguous sequences “*N*,” low-quality reads, and reads that were mostly repeated bases, such as polyT tracts using Trimmomatic 0.39 (26). The trimmed reads are evaluated with FastQC (27), a Java-based, quality control tool for high-throughput sequence data.

After obtaining clean reads and quality checks, RF transcriptome assembly was conducted with Trinity software v2.8.6 with default parameters (28, 29). Only assembled transcripts with lengths of >300 bp were included in further analysis.

Simultaneously, the raw reads were mapped to the *B. bubalis* genome (UOA_WB_1 accessed from https://www.ncbi.nlm.nih.gov/assembly/GCF_003121395.1/) with TopHat2 v2.0.13 (30), using Bowtie2 v2.2.6 (31) as the underlying aligner. Reads aligning to the UOA_WB_1 build were quantified, which disregarded any read/read pair that aligned to more than one location or more than one gene at a single location.

### Differential expression analysis

For the RF assembly results, differential transcript expression for different datasets was calculated using an exact test in the Bioconductor R package (32) edgeR [Empirical Analysis of Digital Gene Expression Data in R (33)]. We used RSEM [RNA-Seq by Expectation-Maximization (34)] to generate read counts for the optimized assembled transcriptome to input into edgeR. EdgeR normalizes raw input data using a trimmed mean of M-values (TMM), and transcripts with artificially low counts (<1 count across all samples) after normalization were excluded before differential expression analysis was completed. The transcript level was quantified in terms of Fragments Per Kilobase of transcript per Million mapped reads (FPKM). Differential expression (DE) was detected using the edgeR Bioconductor package with a log_2_ fold change threshold of 2.

Differential expression analysis for the *B. bubalis* RB assembly was conducted using the Cufflink analysis tool between different samples of the same traits in pairs (high and low yielding). DE genes with log_2_ fold change of ≥2.0, an adjusted *p*-value (padj) of <0.05, and an adjusted FDR of <0.05 were subjected to further analyses.

### Functional annotation of genes

Functional analysis of the DEGs was performed using Blast2GO v 2.5 (35). Blast2GO is a gene ontology-based annotation tool and found to be effective in the functional characterization of sequence data. The DEGs homologous with annotated proteins in the nr database were selected for functional characterization based on the maximum *E*-value (1E-3) and the minimum alignment size (HSP length 33) using BLASTX. The DEG sequences were then categorized according to the GO vocabularies into three categories, i.e., molecular function, biological process, and cellular component. The distribution of GO terms was analyzed at level 2 of the Directed Acyclic Graphs. Annotated DEGs were analyzed for pathway identification using KEGG.

### SSR mining

Simple sequence repeats were identified using the MISA tool from the DEGs. The chromosome-wise distribution of DEGs, SSRs, and SNPs [extracted from DDRAD sequence data (4)] was graphically mapped using the CIRCOS (version 0.69) visualization tool.

## Results

Analysis of our data with both cattle and water buffalo reference assemblies gave varied results for differential expression and annotation among different traits, viz., milk volume, age at first calving, post-partum cyclicity, and feed conversion efficiency.

### Assembly benchmarking

A total of 857 million raw reads were generated (428,671,371 paired-end reads) by Illumina sequencing of the 16 *B. bubalis* samples for four traits, viz., milk volume, age at first calving, post-partum cyclicity, and feed conversion efficiency, with an average of ~26.7 million reads per sample. From these, ~773 million reads (90.7%) were attained after removing the adapters and trimming for quality. These post-cleaning reads passed the minimum quality standards of FastQC.

After read filtering, clean reads were assembled into 488,811, 86,054, 451,596, and 451,596 contigs, reaching a total length of 482,785,524 bp, 81,971,386 bp, 431,765,546 bp, and 529,684,800 bp for “milk volume,” “age at first calving,” “post-partum cyclicity,” and “feed conversion efficiency” traits, respectively. The average length of assembled contigs was 406, 383, 390, and 405 bp and N50 of 1,606, 1,728, 1,588, and 1,588 bp for “milk volume,” “age at first calving,” “post-partum cyclicity,” and “feed conversion efficiency” traits, respectively.

During the course of the abovementioned analysis, the *B. bubalis* genome was sequenced in 2019, and we proceeded with the RB assembly to compare the two assembly results. To evaluate the assembly quality, we mapped the Illumina clean reads on the water buffalo reference genome (UOA_WB_1, Accession GCA_003121395.1). Approximately 91.22% of the paired-end reads were mapped properly.

### Differential expression analysis

A total of 14,201 and 279 DEGs were identified corresponding to RF and RB assembly, respectively, in four traits. The DEGs identified through RF assembly were more as compared to RB assembly. The number of upregulated genes was 7,190 and 126 while the downregulated genes were 7,011 and 153, respectively, expressed in RF and RB assembly (Table 1).

**Table 1:** Number of differentially expressed genes in reference-free (RF) assembly and reference-based (RB) assembly.

**Traits/genes**	**Reference-free (RF) assembly**		**Reference-based (RB) assembly**	
	**Up-regulated**	**Down-regulated**	**Up-regulated**	**Down-regulated**
Milk volume (trait 1)	2,020	2,206	29	25
Age at first calving (trait 2)	726	525	37	62
Post-partum cyclicity (trait 3)	2,970	2,498	15	22
Feed conversion efficiency (trait 4)	1,474	1,782	45	44

**Figure 2:**
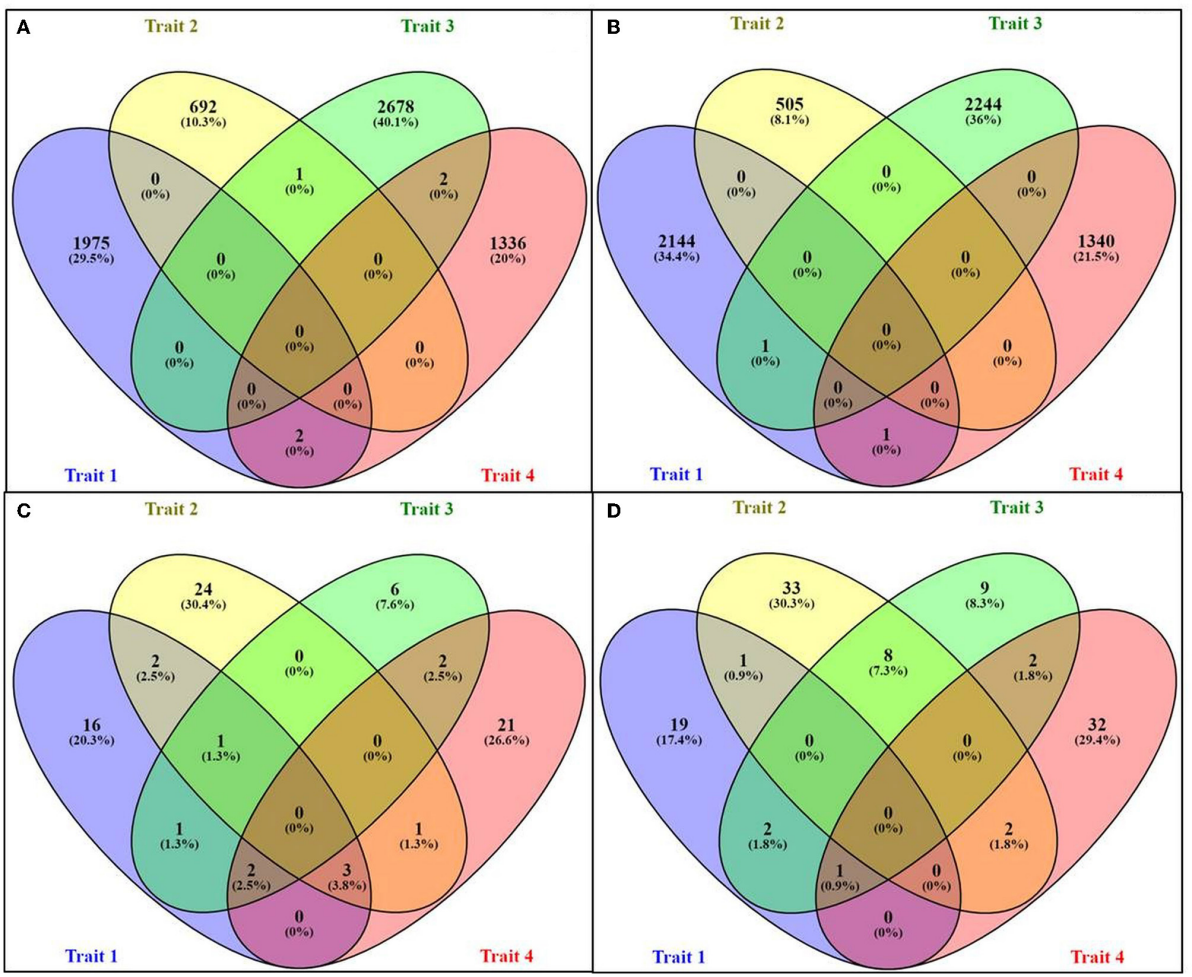
Venn diagram representing the number of upregulated and downregulated genes for different traits: **(A)** upregulated genes in RF assembly, **(B)** downregulated genes in RF assembly, **(C)** upregulated genes in RB assembly, and **(D)** downregulated genes in RB assembly.

While making the comparison among the RF upregulated genes across all four traits, only two common DEGs were common between “milk volume” and “feed conversion efficiency,” and two DEGs were common between “post-partum cyclicity” and “feed conversion efficiency”. A single gene was common between “age at first calving” and “post-partum cyclicity” (Figure 2A). One DEG was found to be common among “milk volume,” “post-partum cyclicity,” and “feed conversion efficiency” traits (Figure 2B).

No common genes were identified among the four traits. A higher number of common DEGs were found in upregulated and downregulated categories through RB assembly. In total, 3.8% of all DEGs were upregulated in “milk volume,” “age at first calving,” and “feed conversion efficiency” traits, which were maximum. For traits, viz., “milk volume,” “age at first calving,” and “post-partum cyclicity,” only two common upregulated DEGs were identified in the RB approach (Figure 2C). There were eight downregulated DEGs (7.3%) common in “age at first calving” and “post-partum cyclicity” (Figure 2D), fairly indicating the level of epigenetic regulation with respect to different traits either through DNA methylation and low expression of mRNA or demethylation.

### Gene annotation

**Figure 3:**
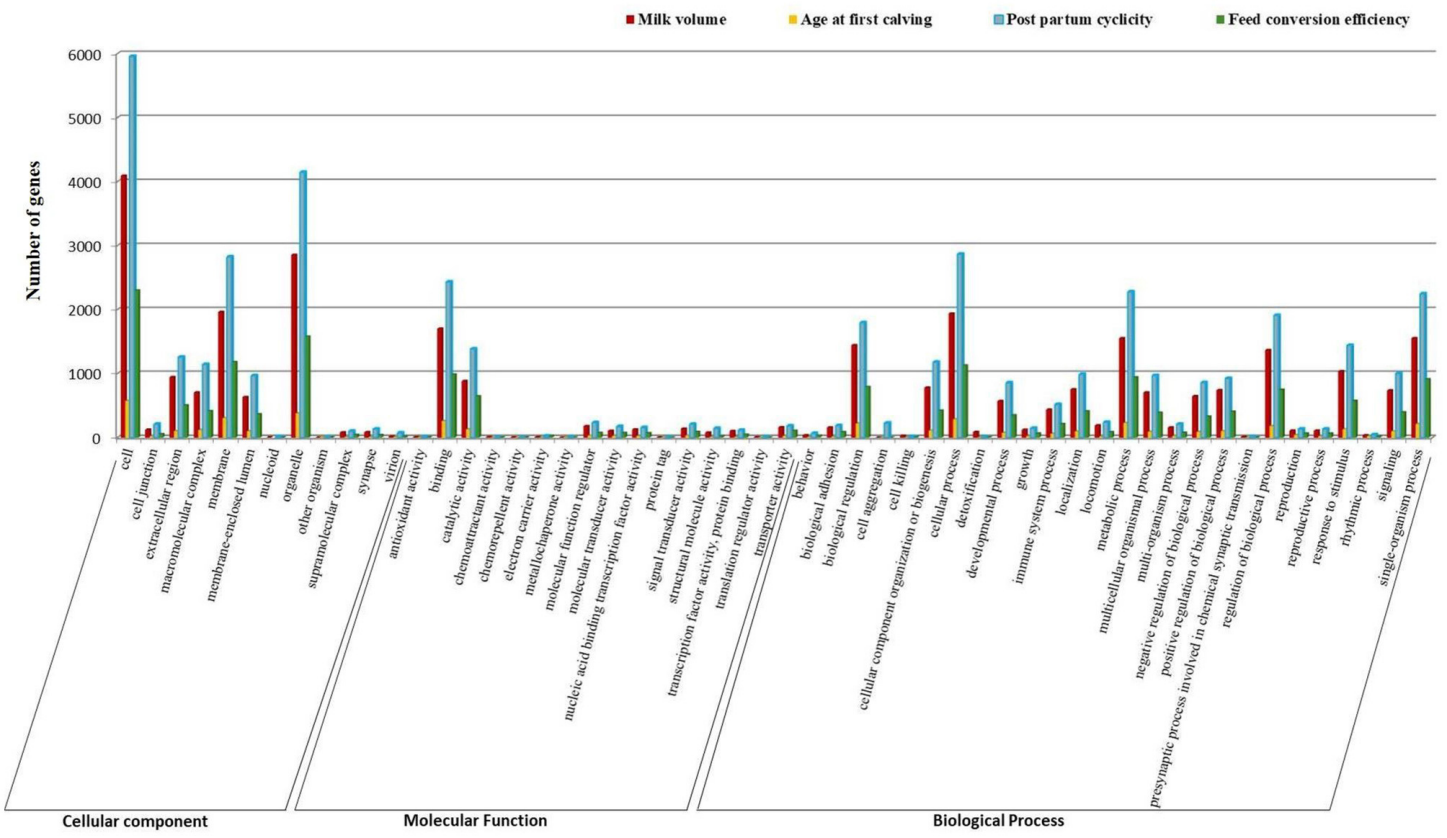
Classification of gene ontology terms in reference-free assembly.

**Figure 4:**
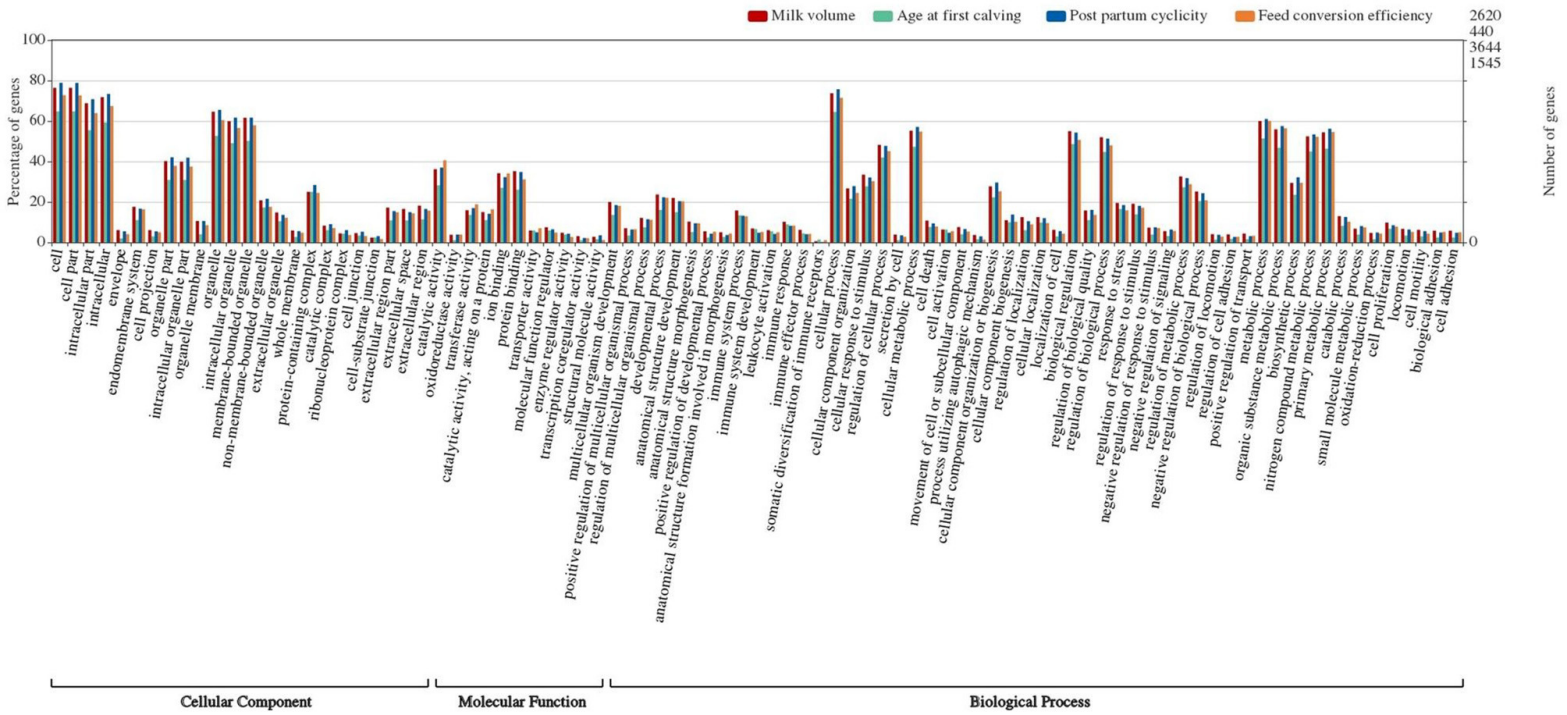
Classification of gene ontology terms in reference-based assembly.

Gene ontology (GO) terms of identified genes (RF) were obtained using the BLAST2Go v 2.5 tool. The study revealed that one or more GO terms, viz., 30,290, 4,228, 43,142, and 17,097, were assigned to genes for milk volume, age at first calving, post-partum cyclicity, and feed conversion efficiency traits, respectively. GO enrichment analysis classifies gene ontology terms into three broad categories, namely, cellular component, molecular function, and biological process (Figure 3). The binding function (GO: 0005488) was the most represented GO term in the molecular function category followed by cell and its part (GO: 0005623, GO: 0044464) and organelle and its part (GO: 0043226, GO:0044422) as cellular component terms for all the traits. Prominent GO terms that emerged from the RF assembly were similar to the classified terms that emerged from the RB assembly as 2,620 for milk volume, 440 for age at first calving, 3,644 for post-partum cyclicity, and 1,545 for feed conversion efficiency traits (Figure 4).

### Variant distribution

**Figure 5:**
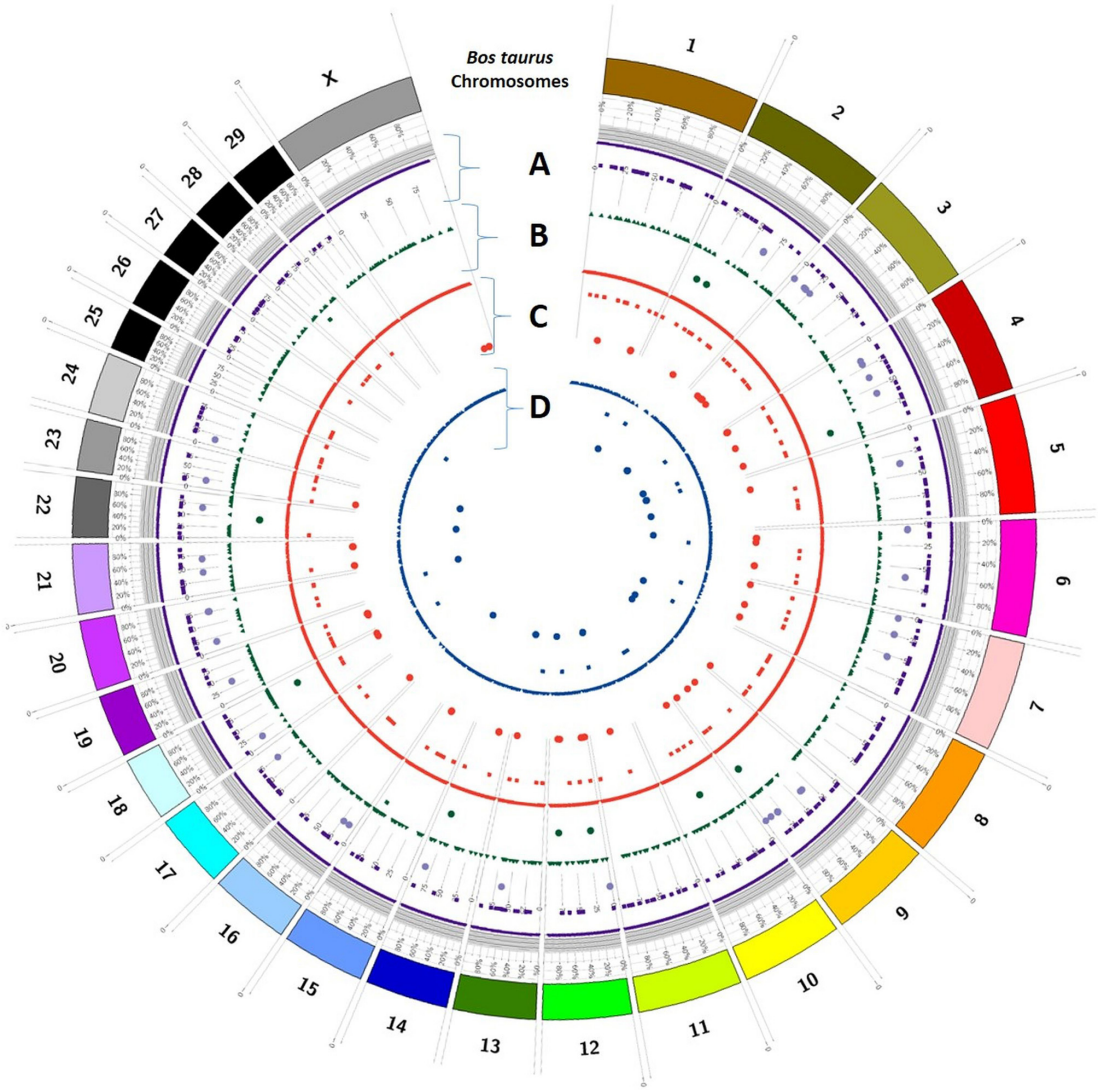
Trait-wise distribution of DEGs, corresponding SNPs, and SSRs mapped on the *Bos taurus* chromosomes using CIRCOS [**(A)** milk volume; **(B)** age at first calving; **(C)** post-partum cyclicity; **(D)** feed conversion efficiency].

**Figure 6:**
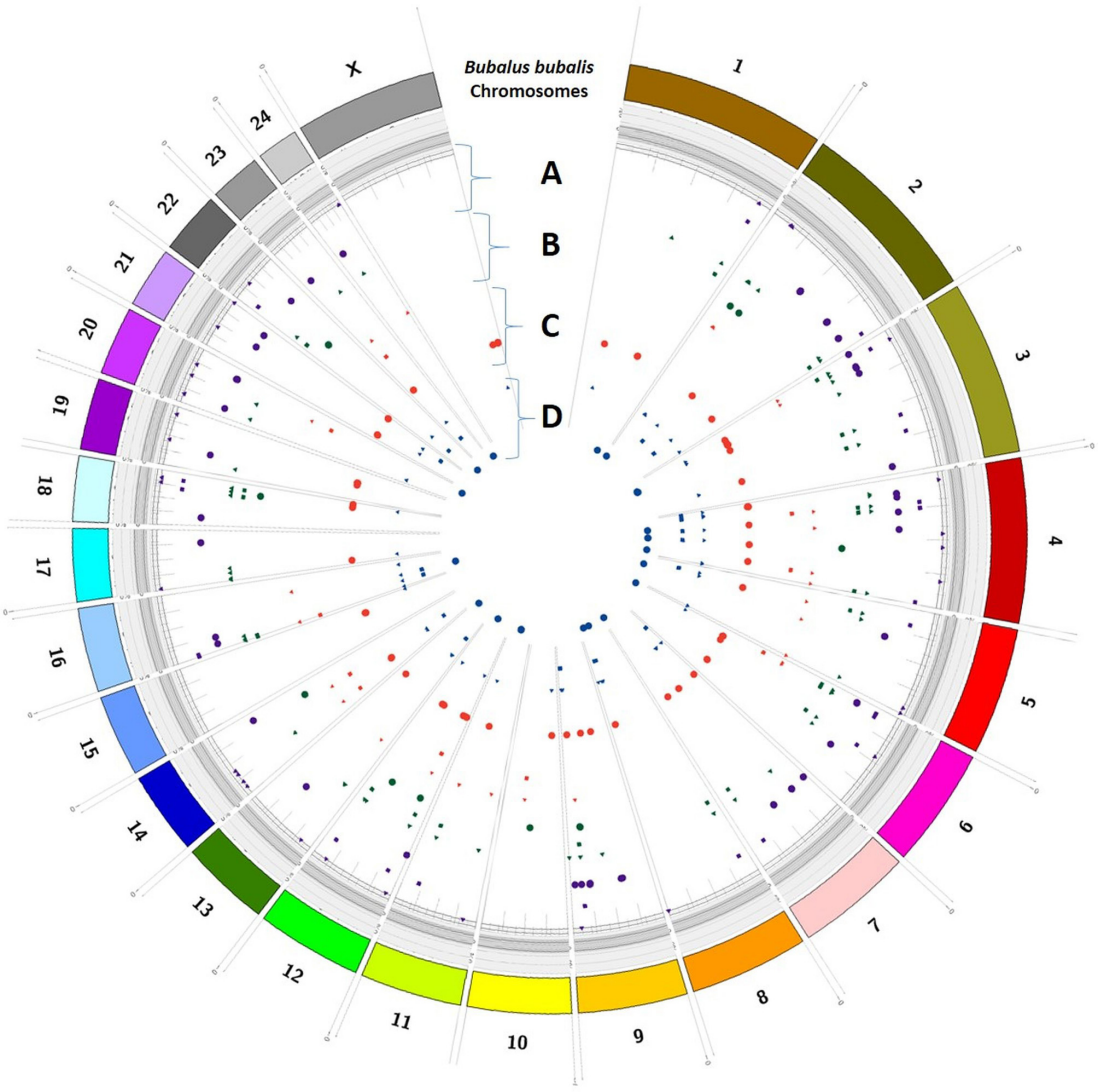
Trait-wise distribution of DEGs, corresponding SNPs, and SSRs mapped on the *Bubalus bubalis* chromosomes using CIRCOS [**(A)** milk volume; **(B)** age at first calving; **(C)** post-partum cyclicity; **(D)** feed conversion efficiency].

Trait-wise distribution of three identified elements as DEGs, SSRs, and SNPs was mapped on the *Bos taurus* genome (Figure 5) and *B. bubalis* genome (Figure 6) using the CIRCOS tool. This depicts the comparative view of the chromosomal-wise distribution of identified elements in various traits, viz., milk volume, age at first calving, post-partum cyclicity, and feed conversion efficiency. In the RB approach, there are 10,114 SSRs across all four traits, viz., milk volume (3,185), age at first calving (529), post-partum cyclicity (3,828), and feed conversion efficiency (2,572). Mononucleotide SSRs are 6,061 followed by 1,779 for dinucleotide SSRs, 1,417 for trinucleotide SSRs, 55 for tetranucleotide SSRs, 25 for pentanucleotide SSRs, and only two hexanucleotide SSRs. These identified SSRs were further filtered based on their chromosomal locations within the identified DEGs and SNPs. Figure 6 shows the mapping of these resulted in 161 SSRs.

## Discussion

The selection programs of domestic animals will be strengthened by a detailed grasp of the genetic variation underlying complex phenotypes (36) as analyzed in this study. The current study compared the DEGs identified through RF and RB assembly approaches and shows their association with the buffalo traits considered in this study. An attempt was also made to show the chromosomal distribution of DEGs, SNPs, and SSRs in respect of all four considered traits, viz., milk volume, age at first calving, post-partum cyclicity, and feed conversion efficiency of buffalo using the CIRCOS tool (Figures 5, 6). These maps depict that the maximum number of DEGs are found in post-partum cyclicity, followed by milk volume and feed conversion efficiency in the RF approach, and age at first calving has the maximum number of DEGs, followed by feed conversion efficiency and milk volume in the RB approach (Table 1).

Phosphatidylinositol 3,4,5 trisphosphate five phosphatase was identified as a common gene that was deregulated for functional diversity in two traits i.e., age at first calving and post-partum cyclicity, indicating the importance of the cell cycle progression in these traits and perhaps regulating the development of embryos (37, 38) (Supplementary Table S1). The FKBP4 gene is responsible for reproductive traits (39, 40). The presence of calcium channel, voltage-dependent, alpha-2/delta subunit 1 (CACNA2D1) gene variant in respect of post-partum cyclicity and feed conversion efficiency traits in our study is crucial for its role in excitation–contraction coupling in neurons, glial cells, and muscle cells (41) (Supplementary Table S1).

The key genes, inositol 1,4,5-triphosphate receptor (ITPR), branched-chain amino acid transaminase (BCAT), and other immunity-related genes, such as T-cell surface glycoprotein and AP-1 transcription factor, are identified as differential genes in our study that are mainly associated with all traits (42). Our study also shows that the identified candidate genes such as growth and hormone receptors, ribosomal proteins, sterol regulatory protein, and GTPase are associated with milk volume as reported earlier by Surya et al. (43), Crisa et al. (44), Wickramasinghe et al. (45), Ma and Corl (46), and Lemay et al. (47), respectively, because of their involvement in histone modification, epidermal differentiation, cell adhesion, and cytoskeletal architecture (48). RNA binding FOX, transmembrane proteins, RNA binding proteins, cytosolic peptidase, and cell adhesion receptor genes were pertinent to age at first calving. These genes are primarily involved in cell proliferation, differentiation, adhesion, the mitotic cycle, DNA replication, RNA transcription, and apoptosis (49) (Supplementary Table S1).

As compared with RF, the RB assembly showed more common genes among the traits. There were a total of 28 genes that were common between different traits in RB compared to RF with only seven common genes (Figure 2). The genes, namely, sphingosine-1-phosphate (SIP), somatostatin (50, 51), BOLA class 1 histocompatibility antigen (52–54), interferon-stimulated/induced protein, and SRY-box transcription factor (55) have maximum homology when aligned with *B. bubalis* genome (UOA_WB_1, Accession GCA_003121395.1; Supplementary Table S2). The gene S1P is a bioactive lipid that acts through cell-surface receptors to promote cell signaling and causes a variety of cellular responses to help in developing immunity against diseases (50, 51). The major histocompatibility complex, such as BOLA class 1 histocompatibility antigen, present in all mammalian species, is crucial for the immune system's development (54). This BOLA histocompatibility complex shows resistance to infectious diseases along with governing the milk volume (52, 53) (Supplementary Table S2). The genes governing disease resistance or susceptibility will positively affect milk productivity.

An immediate defense against viral infection identified in our study is provided by interferon-stimulated genes (ISGs) whose expression is induced by interferon signaling (56). These ISGs also act as potential biomarkers to avoid the occurrence of certain diseases in mammals and eliminate the incidence of adverse reactions to avoid the risk of further damage to the animals (57). This will help in increasing overall productivity that may be either due to an increase in milk volume or due to the application of feed conversion efficiency (Supplementary Table S2).

Post-partum cyclicity-related genes are myosin-related proteins, ribosylhydrolases, and cell adhesion receptors (58) that play a significant role in the growth and immunity of the bovine family. Furthermore, this study also identifies some vital genes (ligand-dependent nuclear receptors such as carboxylase and DLG2) related to feed conversion efficiency for regulating energy homeostasis, apoptosis, immune response, and cell growth in young heifers (59–61) (Supplementary Table S2).

In this study, the enriched GO terms revealed were related to milk production, reproduction, immunological response, and susceptibility/resistance to diseases. GO terms related to milk volume are associated with the biosynthesis of glycoproteins, fatty acids, glycerolipids, sterols, and other biological processes, such as oxidative stress, metabolic processes, transporter activity, divalent metal ion transport, calcium channel activity, acetyltransferase activity, and mRNA processing (Figures 3, 4). This confirms the importance of these processes in lactogenesis (62). The GO terms related to cellular component organization (GO:0071840), cell enzyme activity, or gene expression in response to stimulus (GO:0050896), regulation of biological functions (GO:0050789, GO:0048518, and GO:0048519), single-organism process (GO:0044699), and cell death (GO:0001906) govern physiological processes in animals. These terms are related to age at first calving and feed conversion efficiency (22). Important GO terms stimuli (GO:0050896), regulation of biological quality (GO:0065008), and molecular function (GO:0065009) are related to milk volume and feed conversion efficiency. Genes categorized under cell and cell-part localization (GO:0005623, GO:0030054, and GO:0044464) are prominent in all the considered four traits and are found to regulate different biological processes, viz., trans-membrane transport, regulation of signal transduction, milk production, and chemical transmission (49, 63) (Figures 3, 4). The gene condensing complex subunit 2 (Q3MHQ) encoded by GO term GO:0065007 is linked with the feed efficiency trait. The gene Q3MHQ regulates cell division and improves growth development in an animal by converting interphase chromatin into mitotic chromosomal condensation and is interestingly linked to metabolic pathways involved in feed conversion efficiency (64, 65).

## Conclusion

In this study, an attempt has been made to compare reference-free and reference-based approaches to identify and annotate differentially expressed genes in *B. bubalis* for four important traits, viz., milk volume, age at first calving, post-partum cyclicity, and feed conversion efficiency. Reference-free (RF) *de novo* transcriptome assembly approach is commonly used due to the non-availability of a complete reference genome with the high-quality genetic information of particular species. In this study, the RF approach identified 7,190 upregulated genes and 7,011 downregulated genes, whereas the RB approach identified 126 and 153 genes, respectively. The number of gene ontology terms associated with identified DEGs for the traits under consideration—milk volume, age at first calving, post-partum cyclicity, and feed conversion efficiency—were 30,290, 4,228, 43,142, and 17,097 for the RF approach, compared to 2,620, 440, 3,644, and 1,545 terms for the RB approach. The identified genes and GO terms will establish a sound base for biological postulates which will further improve future animal breeding programs to enhance animal productivity.

## Data availability statement

The original transcriptome data presented in the study are publicly available. This data can be found here: NCBI Bioproject, accession number PRJNA934134.

## Ethics statement

The animal study was reviewed and approved by Institute Animal Ethics Committee (IAEC) at ICAR-Central Institute for Research on Buffaloes, Hisar, India.

## Author contributions

PS, AJ, AB, and IS conducted the experiments. JB and SY did data analysis. DM, JB, KC, and HA conceptualized the study and manuscript. All authors reviewed and approved the final manuscript.
